# Indole-3-carbinol in vitro antiviral activity against SARS-Cov-2 virus and in vivo toxicity

**DOI:** 10.1038/s41420-022-01280-2

**Published:** 2022-12-15

**Authors:** Federica Centofanti, Tonino Alonzi, Andrea Latini, Paola Spitalieri, Michela Murdocca, Xiaodong Chen, Weibo Cui, Qianwen Shang, Delia Goletti, Yufang Shi, Andrea Duranti, Carlo Tomino, Michela Biancolella, Federica Sangiuolo, Maria Rosaria Capobianchi, Suresh Jain, Giuseppe Novelli, Pier Paolo Pandolfi

**Affiliations:** 1 Department of Biomedicine and Prevention, Tor Vergata University of Rome, 00133 Rome, Italy; 2 Translational Research Unit, National Institute for Infectious Diseases, IRCCS, Rome, Italy; 3 Wuxi Sinotide New Drug Discovery Institutes, Huishan Economic and Technological Development Zone, 1699 Huishan Boulevard, Wuxi, Jiangsu China; 4 Department of Experimental Medicine, Tor Vergata Oncoscience Research Centre of Excellence, TOR, University of Rome Tor Vergata, 00133 Rome, Italy; 5 The First Affiliated Hospital of Soochow University and State Key Laboratory of Radiation Medicine and Protection, Institutes for Translational Medicine, Soochow University, 199 Renai Road, Suzhou, 215123 Jiangsu China; 6 Department of Biomolecular Sciences, University of Urbino Carlo Bo, 61029 Urbino, PU Italy; 7 Scientific Direction - IRCCS San Raffaele, 00166 Rome, Italy; 8 Department of Infectious Tropical Diseases and Microbiology, Sacro Cuore Don Calabria Hospital I.R.C.C.S., Negrar di Valpolicella, Verona, Italy; 9 Intonation Research Laboratories, Hyderabad, India; 10 IRCCS Neuromed, Pozzilli, IS Italy; 11 Department of Pharmacology, School of Medicine, University of Nevada, Reno, NV 89557 USA; 12 William N. Pennington Cancer Institute, Renown Health, Nevada System of Higher Education, Reno, NV 89502 USA

**Keywords:** Preclinical research, Medical research

## Abstract

The effects of indole-3-carbinol (I3C) compound have been described deeply as antitumor drug in multiple cancers. Herein, I3C compound was tested for toxicity and antiviral activity against SARS-CoV-2 infection. Antiviral activity was assessed in vitro in both in VeroE6 cell line and human Lung Organoids (hLORGs) where I3C exhibited a direct anti-SARS-CoV-2 replication activity with an antiviral effect and a modulation of the expression of genes implicated in innate immunity and inflammatory response was observed at 16.67 μM. Importantly, we further show the I3C is also effective against the SARS-CoV-2 Omicron variant. In mouse model, instead, we assessed possible toxicity effects of I3C through two different routes of administration: intragastrically (i.g.) and intraperitoneally (i.p.). The LD50 (lethal dose 50%) values in mice were estimated to be: 1410 and 1759 mg/kg i.g.; while estimated values for i.p. administration were: 444.5 mg/kg and 375 mg/kg in male and female mice, respectively. Below these values, I3C (in particular at 550 mg/kg for i.g. and 250 mg/kg for i.p.) induces neither death, nor abnormal toxic symptoms as well as no histopathological lesions of the tissues analysed. These tolerated doses are much higher than those already proven effective in pre-clinical cancer models and in vitro experiments. In conclusion, I3C exhibits a significant antiviral activity, and no toxicity effects were recorded for this compound at the indicated doses, characterizing it as a safe and potential antiviral compound. The results presented in this study could provide experimental pre-clinical data necessary for the start of human clinical trials with I3C for the treatment of SARS-CoV-2 and beyond.

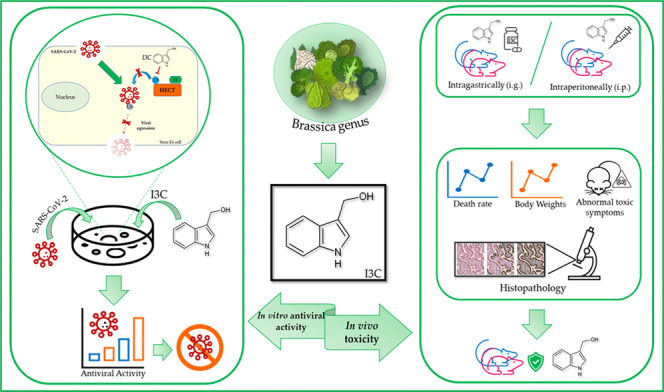

## Introduction

**Fig. 1:**
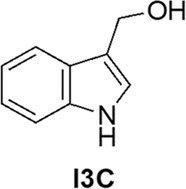
I3C chemical structure. Chemical structure representation of indole-3-carbinol (I3C).

Indole-3-carbinol (I3C, Fig. 1) is an interesting compound that is much sought-after targets due to its broad biological properties since it is used as an antitumor agent [1].

I3C is a natural indole carbinol phytochemical derived by hydrolysis from glycobrassicin by plant or bacterial myrosinase produced in cruciferous vegetables of the *Brassica* genus such as cabbage, broccoli and Brussels sprouts, and is able to activate multiple antiproliferative cascades [2, 3].

For decades, it has been widely explored regarding potential roles in several human cancer types [4, 5] (i.e., melanoma, breast, prostate, lung, colon, leukaemia, and cervical cancer [6–16]) for its chemopreventive action on cancer in pre-clinical models and also for its promising effectiveness in clinical trials [17]. Many studies showed that I3C is involved in different cellular mechanisms, including transcriptional, enzymatic, metabolic and cell signalling processes. In particular, I3C can induce the suppression of cell cycle progression, block cancer cell migration, the promotion of apoptosis, and the inhibition of tumour growth [18–20]. Although I3C interacts with different pathways, the exact mechanisms by which it influences human cells have not yet been fully understood. It has been proposed that I3C may mediate its anti-proliferative effects in cancer cells by directly interacting with different classes of target proteins with enzymatic activities. In particular, it has been demonstrated that the I3C and its synthetic derivatives are potent natural inhibitors of HECT family member of E3, suggesting the potential importance of I3C in developing highly potent and stable anti-cancer molecules [21].

Since I3C impacts on many cellular mechanisms, it is not surprising to find proposed applications in the treatment or prevention of other toxicities related illness, organ dysfunctions or other cancer types (other than that of breast, ovarian, prostate, lung, liver and colon). It has been investigated for its application in neurotoxicity, ionizing radiation, thyroid disease, endometriosis, Epstein-Barr viral Burkitt’s lymphoma, papilloma viral-dependent cancers, cervical dysplasia/cancer and metabolic syndrome [17, 22–30].

In January 2020, COVID-19, an infectious disease caused by the Severe Acute Respiratory Syndrome Coronavirus 2 (SARS-CoV-2) rapidly became pandemic worldwide [31]. This pandemic is ongoing, and the global number of confirmed SARS-CoV-2 cases continues to rise. SARS-CoV-2 is a novel coronavirus belonging to the Beta-coronavirus genus [32]. Since the pandemic broke out, international research laboratories, together with pharmaceutical and biotech companies, are working in an unprecedented manner at extraordinary speed to find and evaluate drugs, vaccines and other solutions aimed at decreasing hospital admissions and helping heal patients and support recovery.

Nowadays, the epidemiological trend of SARS-CoV-2 does not allow to hypothesize a rapid disappearance of the disease and there are no data available on the possible protection spectrum and immunity time conferred by the vaccines currently being used or available and by those under development [33, 34]. For these reasons, it is vitally important to have molecules that can reduce the infection burden and the severity of lesions in individuals with SARS-CoV-2 infection [35–37]. To identify new drug candidates, different approaches are being explored, starting with artificial intelligence and in vitro and in vivo studies, and aimed at identifying molecules capable of blocking the entry of the virus into cells, its intracellular replication, and cell egress. Several lines of research suggest that SARS-CoV-2 neutralizing antibodies (nAbs) that bind directly to the virus’s spike glycoprotein and inhibit entry into host cells have therapeutic potential [38]. However, monoclonal antibodies currently approved for clinical use clinic either fail to neutralize the Omicron virus, with its mutated spike protein, or demonstrate significantly reduced neutralizing efficiency [39].

Recently, I3C displayed potent anti-SARS-CoV-2 effects [40]. We demonstrated that HECT proteins are involved in SARS-CoV-2 pathology, physically interacting with and ubiquitylating the SARS-Cov-2 spike protein. We showed that some members of the HECT family are expressed in greater quantities in the human cells of infected subjects and in the mouse models of COVID-19. Moreover, we found that several rare variants in the *NEDD4 E3 ubiquitin protein ligase* (*NEDD4*) and *WW domain containing E3 ubiquitin protein ligase 1* (*WWP1*) genes are associated with severe cases of COVID-19, when compared to asymptomatic controls [40]. In addition, we proved that I3C is able to block SARS-CoV-2 viral egression in Vero E6 model through the inhibition of HECT proteins implicated in Covid-19 pathology [40]. The importance of E3 ligases in the ubiquitination of some viral proteins has recently been confirmed by combined multiomics studies which have allowed to demonstrate the ability of SARS-CoV-2 not only in the remodelling of the innate immunity, but also in promoting viral infection, by hijacking specific processes of ubiquitination [41]. All these data suggest the potential use of I3C as antiviral in clinical trials for patients with COVID-19.

Here, we assessed I3C pharmacological potential, investigating the toxicity and antiviral effect of I3C in in vitro and in vivo models.

## Results

### I3C antiviral activity in vitro

**Fig. 2:**
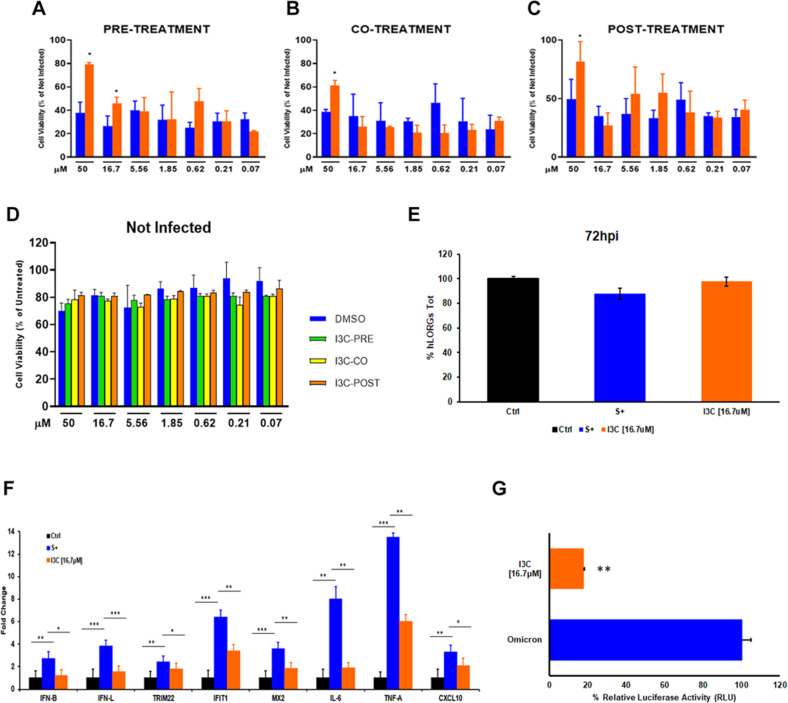
I3C antiviral activity in vitro*.* Cells were treated with different doses of I3C (orange bar) or DMSO (blue bar) 1 h before (**A**), together (**B**) and 1 h after (**C**) of SARS-CoV-2 infection (MOI = 0.001). **D** Cells treated as in (**A**–**C**) were left uninfected to evaluate cell toxicity. The results were evaluated 72 h post-treatment by setting the uninfected and untreated control cells as 100% and the remaining values represented as a relative value. Data are expressed as mean ± SD (*n* = 9) of three independent experiments performed in triplicated. Results were analysed using Graph Pad Software (GraphPad Prism 9). Statistically significant differences between DMSO and I3C are represented as *p < 0.05 determined using the Wilcoxon test. **E** Percentage of hLORGs tot reformed after 72 h post infection (hpi) with VSVpp.SARS-2-S D614G variants. *n* = 25–30 fields from three biological replicates and two different observers. **F** Expression of immunity-related genes in hLORGs 72 h post-treatment, with (S+) and without (Ctrl) spike protein of VSVpp.SARS-2-S and following treatment with I3C at 16.7 µM. Bar graph shows expression of immune response genes and cytokines, quantified by qRT-PCR at 72 h post-treatment (*n* = 6). *p < 0.05, **p < 0.01 and ***p < 0.001 by one-way ANOVA test. **G** Transduction efficiency was quantified by measuring virus-encoded luciferase activity in cell infected after 72 h post I3C treatment with Pseudo type Lentivirus SARS-2-Omicron. Data are expressed as the percentage of infection and the average data (*n* = 6) from three biological replicates and reported as mean ± standard error of the mean (SEM). Each biological replicate has been performed in duplicate. **p < 0.01 by Student’s t-test.

We evaluated at first the impact of I3C on the antiviral activity during SARS-CoV-2 infection in Vero E6 cells regardless the time of treatment. We treated the cells with I3C using a 3-fold decreasing concentration scale ranging between 50 and 0.069 μM based on using three different treatment protocols: (i) 1 h before SARS-CoV-2 infection (pre-treatment); (ii) concomitantly with the infection (co-treatment) and (iii) 1 h after infection (post-treatment). The drug was then added at two-time points after (24 and 48 h) SARS-Cov-2 infection (MOI = 0.001). Antiviral activity was evaluated 72 h post-infection, when the viral-induced cytopathic effects (CPE) were evaluated. We observed that the pre-treatment protocol with I3C significantly reduced the SARS-CoV-2-induced CPE both at 50 [whose concentration we know to be partially toxic to cells [40]] and 16.67 μM, when compared to DMSO-treated cells (*p < 0.05). The reduction was lost at lower I3C concentrations (Fig. 2A).

On the other hand, in I3C co- and post-treatment protocols, we observe a statistically significant reduction only at 50 μM (*p < 0.05), while no significant differences were observed in the other I3C concentrations, when compared to DMSO-treated cells (Fig. 2B, C, respectively). Moreover, to see the direct effect on cells, we also evaluated the effect of I3C at the used concentrations on the cell viability without any infection, but we did not observe any different between I3C treatment and DMSO control in terms of cell viability (Fig. 2D).

Overall, these data demonstrated that the I3C pre-treatment protocol exerts a direct anti-SARS-CoV-2 activity, and it appears as the best treatment schedule compared to co- and post-treatment protocols, because we observed an antiviral effect at 16.67 μM. In particular, this data highlight that I3C has an antiviral effect also when the cells are already SARS-CoV-2-infected, as measured by CPE-inhibition assay. This is an important finding, further suggesting I3C as a possible repurposing molecule candidate for Covid-19 therapy.

To investigate the effects of I3C pre-treatment protocol on innate immune response and support its efficacy against VSVpp.SARS-2-S, we evaluated the immunity-related genes expression in infected human Lung Organoids (hLORGs) 72 h after I3C treatment at 16.7 µM. First, following the infection and treatment, we went to count the number of organoids that form after 72 h of treatment. In organoids only untreated (Ctrl), infected (S+), and treated and infected with I3C [16.7 μM], we obtained about the same number of hLORGs formed (Fig. 2E), to demonstrate the fact that if I3C was toxic it would not allow the single cells that make-up organoids to self-assembly. Secondly, as a reported in [42], we confirmed, by RT-qPCR, that pseudo-SARS-CoV-2 induced *type I* (*IFNβ*) and *III* (*IFNγ1*) *IFNs* expression, the first protection against viral infections, as well as the expression of interferon-stimulated genes (*IFIT1*, *TRIM22*, and *MX2*), which counteract viral replication, transcription, and translation in infected and uninfected cells and stimulate the adaptive immune response. In addition, we observed that mRNAs levels of pro-inflammatory chemokines and cytokines (*CXCL10*, *IL-6* and *TNF-α*) were significantly upregulated in pseudo-SARS-CoV-2-infected hLORGs (***p < 0.001) (Fig. 2F).

After I3C pre-treatment protocols, we observed a significant decrease in mRNA levels of *type I and III IFNs* in infected hLORGs treated with I3C at a concentration of 16.7 µM compared to infected (S+) hLORGs (*p < 0.05). The hLORGs treated with I3C also showed a significant downregulation of interferon-stimulated genes (*TRIM22*, *IFIT1* and *MX2*) and of pro-inflammatory chemokines and cytokines (*IL-6*, *TNF-α* and *CXCL10*) (*p < 0.05). This downregulation is evident for all genes in hLORGs after 16.7 µM of I3C treatment (Fig. 2F).

We also evaluated I3C pre-treatment protocol at 16.7 µM in infected VeroE6 cell line with Pseudotype Lentivirus SARS-2-Omicron. The antiviral effect of I3C was evaluated by a luciferase assay, which quantifies the ability of virus to infect VeroE6 cells. As observed in Fig. 2G, the capacity of pseudovirus Omicron to infect target cells was strongly inhibited. Specifically, the infection rate of cells treated with I3C was 18% in this pseudotype lineage tested (**p < 0.01) (Fig. 2G). Based on data from the luciferase assay, it can be concluded that the I3C pre-treatment protocol is also able to inhibit the pseudotype lentivirus SARS-2-Omicron infection, demonstrating that pre-treatment protocol is the best treatment schedule with the new emerging SARS-CoV-2 variant.

### Preclinical toxicity in a mouse model

#### Lethal dose (LD50) and toxicity grade of I3C in mice

To determine the short-term adverse effects of I3C on main target organs when administered in a single dose after intraperitoneal (i.p.) and intragastric (i.g.) administration route in mice, we firstly estimate a Lethal Dose (LD50) of I3C in i.p. and i.g. administration and its toxicity grade both for male and female mice using AOT425StatPgm Software based on maximum likelihood with 95% PL Confidence interval. From this analysis, we obtained the following estimated LD50 values for i.g. administration: 1410 mg/kg (95% PL confidence interval is 861.3 to 1680 mg/kg) and 1759 mg/kg (95% PL Confidence interval is 0 to great than 2000 mg/kg) for male and female mice, respectively (Table 1). The estimated LD50 values for i.p. administration were: 444.5 mg/kg (95% PL Confidence interval is 355.3 to 543) and 375 mg/kg (95% PL Confidence interval is 337.8 to 478) for male and female mice, respectively (Table 1).

**Table 1:** Lethal dose (LD50) and toxicity grade of I3C in mice.

Animal information	LD50 (mg/kg)	The upper 95% confidence limit of LD50 (mg/kg)	The lower 95% confidence limit of LD50 (mg/kg)
Male (i.g.)	1410	1680	845.3
Female (i.g.)	1759	2000	0
Male (i.p.)	444.5	543	355.3
Female (i.p.)	375.0	478	337.8

I3C LD50 intragastric (i.g.) and intraperitoneal (i.p.) route administration in male and female mice values based on UDP (User Datagram Protocol) experiment data.

#### Death rate

All mice (male and female) were divided in two groups (i.g. *vs* i.p.) and treated with different doses of I3C: 2000, 1750, 1500, 1000 and 550 mg/kg in a single i.g. administration and 1000, 550, 375 and 250 mg/kg in a single i.p. administration. After I3C dosing, they were then observed individually and periodically for the first 24 h, with particular attention in the first 4 h, and daily thereafter, for a total of 14 days.

**Fig. 3:**
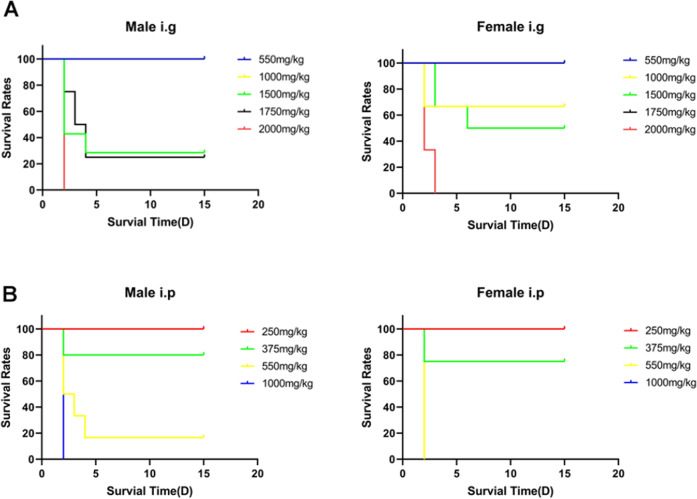
Kaplan–Meier curve after I3C administration in mice. Each point on the curve represents the survival rate of the mice at that point in time. When the X-axis is 0, the follow-up has just begun, and no mice has died, so the survival rate of all groups is 100%. **A** Survival rates after I3C i.g. administration in male (*n* = 3) and female (*n* = 3) mice. **B** Survival rates after I3C i.p. administration in male (*n* = 3) and female (*n* = 3) mice. We observed that single administration of doses higher than 1000 mg/kg (i.g.) and 375 mg/kg(i.p.) to mice determined relevant mortality rate both in male and female mice. Due to the early death of some groups of mice, some group information is not shown on the Fig. (mice died within 4 h from the administration of i.g and i.p.).

As concerned I3C i.g. administration, we observed that single administration of doses higher than 1000 mg/kg to mice determined relevant mortality rate both in male and female mice (Fig. 3A). In particular, all mice (male and female) died within 4 h from the administration of 2000 mg/kg i.g. (data not included in the graph).

Similarly, in I3C i.p. administration, we observed that single-dose administration higher than 375 mg/kg determined a relevant mortality rate, both in male and female mice. All male mice died within 4 h of administration of 1000 mg/kg intraperitoneally while all female mice died within 4 h from the administration of 550 mg/kg intraperitoneally (Fig. 3B).

#### Body weights

All mice were also observed individually for their body weight after I3C i.g. and i.p. dosing at least once during the first 30 min, periodically for the first 24 h, with particular attention in the first 4 h, and daily thereafter, for a total of 14 days.

**Fig. 4:**
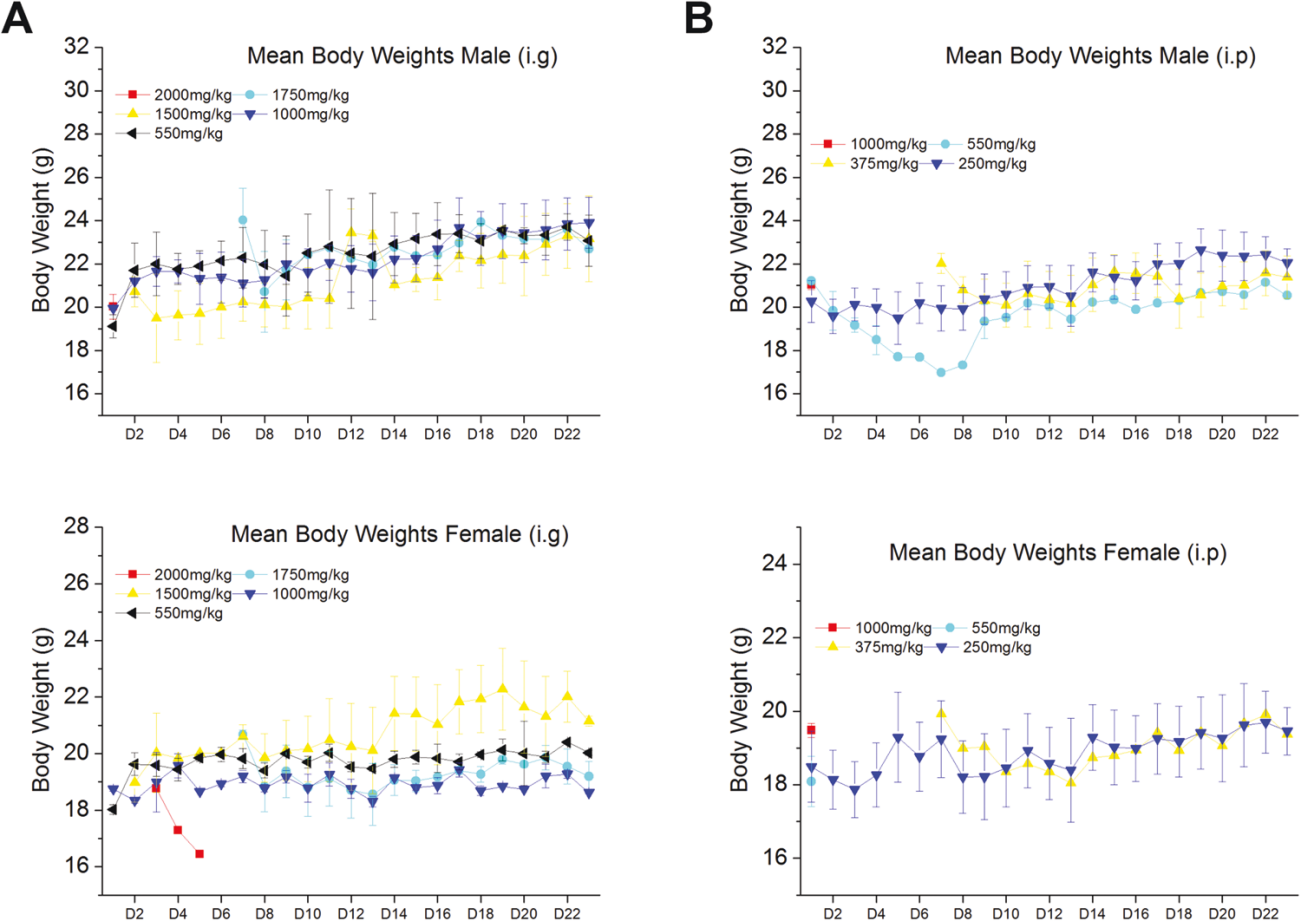
Body weight after I3C administration in mice. **A** Body weight after I3C i.g. administration in male (*n* = 3; upper graph) and female (*n* = 3; lower graph) mice. **B** Body Weight after I3C i.p. administration in male (*n* = 3; upper graph) and female (*n* = 3; lower graph) mice. Some group information not included or gradual disappearance in the graph because they died after administration. Data are expressed as mean ± SD.

We observed that in all mice (male and female) after I3C i.g. administration at different doses, the body weight growth showed a tendency to decrease and then raised slowly when a single dose higher than 1500 mg/kg was administrated (Fig. 4A). Mice treated with 2000 mg/kg are not included in the graph because they died after 4 h, as mentioned in the previous paragraph.

Similarly, when I3C was administrated at different doses i.p., we observed that the body weight growth of mice showed a tendency to decrease and then raised slowly (both in males and females) when a single dose higher than 375 mg/kg was administrated (Fig. 4B).

#### Toxicological results

**Fig. 5:**
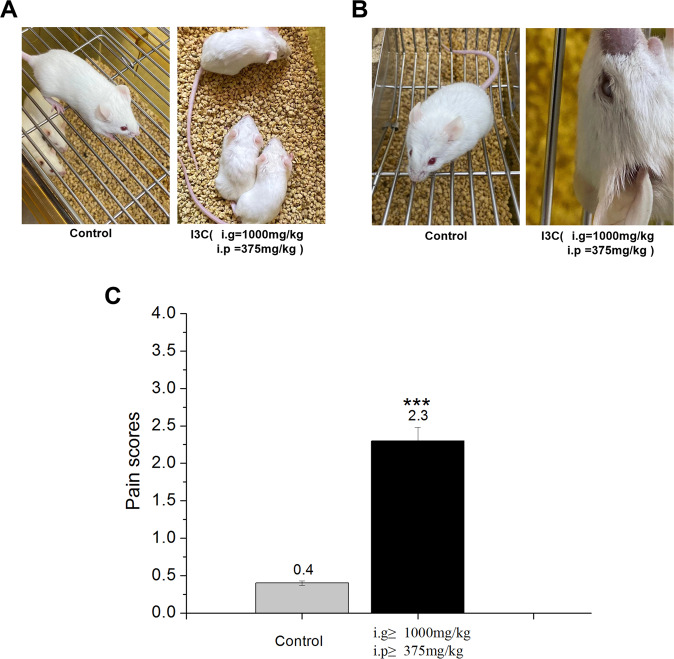
Abnormal toxic symptoms after I3C administration. **A** Piloerection and dorsal hair dull phenomenon (contraction of arrector pili muscle) after I3C administration (*n* = 6) (i.g. ≥ 1000 mg/kg and i.p. ≥ 375 mg/kg) compared to control mice (*n* = 6). **B** Conjunctival opacification, iritis, and conjunctivitis with reduced spontaneous activity after I3C administration (*n* = 6) (i.g. ≥ 1000 mg/kg and i.p. ≥ 375 mg/kg) compared to control mice (*n* = 6). **C** The pain score index of experimental animals increased significantly after I3C administration (Scoring standard of pain index for experimental animals: 0: Normal hair and activity of the experimental animals; 1: part of the hair of the experimental animals was erect and temporarily arched back; 2: the fur of the experimental animals was obviously rough and intermittent arched back; 3: the fur was obviously rough, accompanied by other symptoms such as arched back, slow reaction and behaviour, and even death). Data are expressed as mean ± SD.

Abnormal toxic symptoms were also evaluated after I3C administration. At doses greater than 1000 mg/kg (for i.g. administration) and 375 mg/kg (for i.p. administration) were observed piloerection and dorsal hair dull phenomenon (contraction of arrector pili muscle) appeared in mice (before death) after I3C administration, and spontaneous activity decreased compared to control mice (Fig. 5A). Moreover, mice (some dead mice) developed a phenomenon of conjunctival opacification, iritis, and conjunctivitis with reduced spontaneous activity after I3C administration (i.g. ≥1000 mg/kg and i.p. ≥375 mg/kg) compared to control mice (Fig. 5B). The pain score index of experimental animals showed an increased statistically significant after I3C administration (i.g. ≥1000 mg/kg and i.p. ≥375 mg/kg) compared to control mice (***p < 0.001) (Fig. 5C)

#### Histopathology

The organs of the mice whose survival time is more than or equal to 24 h after exposure, the histopathological examination (heart, liver, spleen, lung and kidney) was carried out in order to obtain relevant toxic information.

**Fig. 6:**
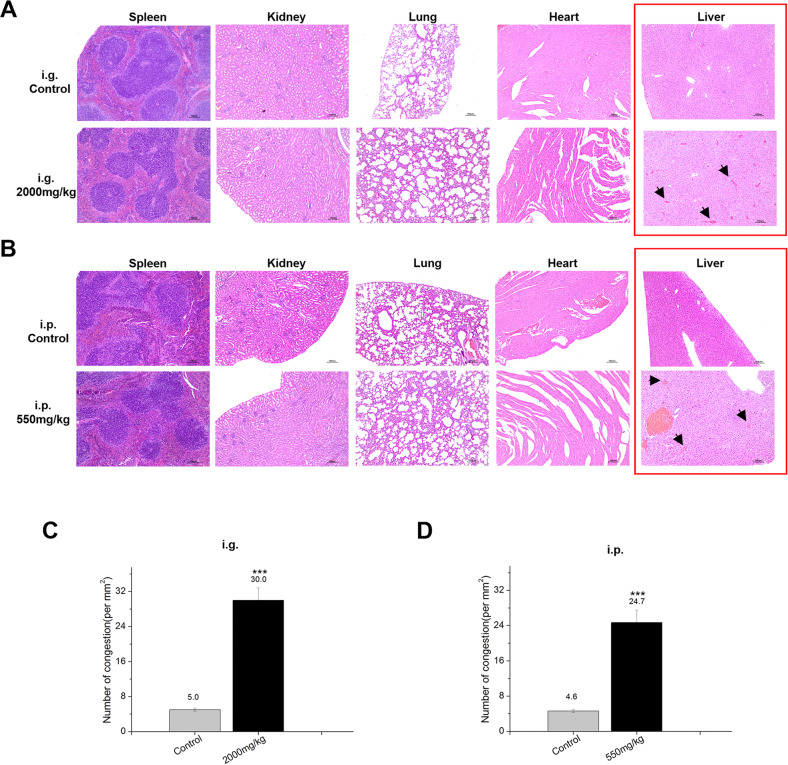
Histopathological examination after I3C administration. **A** Spleen, kidney, lung and heart did not show gross pathological change after I3C i.g. administration at 2000 mg/kg. Only in the liver (red square), we observed congestion areas connected to the blood stasis zone. **B** Spleen, kidney, lung and heart showed no gross pathological change after I3C i.p. administration at 550 mg/kg. We only observed congestion areas in the liver (red square) connected to the blood stasis zone. Congestion was stained with H&E, Black arrows indicate congestion and containing red blood cells. **C** The number of congestions containing red blood cells in each section after I3C i.g. administration was counted. Results are presented as the average number of congestions per mm^2^ as mean ± SD (*n* = 25–30 fields) from three biological replicates and two different observers. **D** The number of congestions containing red blood cells in each section after I3C i.p. administration was counted. Results are presented as the average number of congestions per mm^2^ as mean ± SD (*n* = 25–30 fields) from three biological replicates and two different observers. Statistical significance was determined by one-way analysis of variance, ***p < 0.001.

The histopathological examination of heart, liver, spleen, lung and kidney after I3C i.g. administration reveals that there were no I3C effects on mice given 2000 mg/kg compared to control tissue. There is only congestion in the liver of the mice in the I3C 2000 mg/kg group. The congestion is localized on the central vein. The central vein and the hepatic sinusoids around it were obviously dilated and filled with red blood cells. Some congestion areas are connected to form a blood stasis zone. The hepatocytes in the central area of the lobule atrophied and disappeared, resulting in sparse, scattered and disordered hepatocytes (Fig. 6A).

As concerned I3C i.p. administration, there were no I3C effects in all mice tissues analysed given 550 mg/kg compared to control group. As for i.g. administration, we observed only congestion in the liver of the mice in the I3C 550 mg/kg group compared to control group. The congestion is localized on the central vein. The central vein and the hepatic sinusoids around it were obviously dilated and filled with red blood cells. Some congestion areas are connected to form a blood stasis zone. The hepatocytes in the central area of the lobule atrophied and disappeared, resulting in sparse, scattered and disordered hepatocytes (Fig. 6B). The number of congestions containing red blood cells in each section after both I3C i.g. and i.p. administration was counted. We observed an increased statistically significant of number of congestions containing red blood in both I3C i.g. and i.p. administration (***p < 0.001) (Fig. 6C, D).

## Discussion

Viruses cause a wide spectrum of clinical illnesses, most of which are acute respiratory infections. In most cases, the symptoms of acute respiratory infection are similar for different viruses, though the severity may be variable [43]. Respiratory viral infections represent a significant threat to human health worldwide. SARS-CoV-2 is responsible for the ongoing worldwide pandemic which has already taken more than six million lives [33]. Antiviral drugs are being studied, aimed at inhibiting the replication of the virus; immunomodulatory drugs to attenuate the overactive immune system [44, 45]; neutralizing antibodies to inhibit the virus and help the immune system to clear the infection [38, 46, 47]. To date, about 156 vaccines have been designed and more than 120 clinical trials were underway at that time [48, 49]. However, the duration of protection of the vaccines available today decreases within 3–6 months as evidenced by the rates of breakthrough infections caused by new variants of the virus [34]. Therefore, it urgently needs to have new compounds for its prevention and effective treatment.

Recently, we demonstrated that indole-3-carbinol (I3C), a natural HECT family member of E3 ligases inhibitor, displays potent anti-SARS-CoV-2 effects and inhibits viral egression and can be potentially used as an antiviral drug [40]. So, in this study, we extent the in vitro antiviral activity of I3C against SARS-CoV-2 in Vero E6 cell line. We substantiated that I3C significantly reduce the SARS-CoV-2-induced CPE at 16.67 μM, when compared to DMSO-treated cells and provide evidence that I3C treatment has still antiviral effects when provided at the time of infection or after infection. Specifically, we present novel data demonstrating that I3C displays an antiviral effect also when the cells are already SARS-CoV-2-infected (in particular see the post-treatment protocol; Fig. 2C), as measured by CPE-inhibition assay; while we previously demonstrated that a reduced CPE corresponds to a decreased viral production using either I3C [40] or other compounds [45, 50]. This is an important finding which further supports the notion that I3C is an exciting repurposing molecule candidate for Covid-19 therapy.

SARS-CoV-2 similarly to other RNA viruses causes the immune system to attack its tissues in the form of an autoimmune and autoinflammatory process with an exaggerated release of proinflammatory cytokines and type I interferon (IFN). Therefore, to evaluate the efficacy of I3C as an antiviral, we analysed the cytokine response after infection with SARS-CoV-2 pseudovirus and treatment with I3C on a three-dimensional model of human lung organoids (hLORG) obtained from pluripotent stem cells (iPSC) [42]. We observed a significant reduction in the mRNA levels of IFN-related genes in infected hLORGs treated with I3C at a concentration of 16.7 µM compared to infected (S+) hLORGs (*p < 0.05).

We expanded our analysis to test the possible I3C efficacy to inhibit the very prevalent SARS-CoV-2 Omicron variant. I3C proved to also be effective towards this variant, which is of great relevance in view of the global impact of this variant on human health. These data demonstrate the usefulness of I3C in reducing the efficiency of in vitro infection and provide further evidence that host-targeted antiviral therapy may be beneficial to counter viral resistance and develop broad-spectrum antivirals.

Because I3C is a bioactive compound derived as a compound from *Brassicaceae*, it is perceived as safe, thus increasing interest in its use to prevent several diseases, including Covid-19. However, the effective concentrations of I3C at clinical and preclinical levels and the results associated with the consumption of large amounts remain unclear and warrant further study. For this reason, we evaluated potential adverse in vivo effects in a unique and very comprehensive manner in the mouse as never done before. We evaluated the toxicity through different routes of administration to understand which were the target organs of possible toxicities and assess possible diverse adverse effects due to different administration routes. Indeed, we observed that the liver is the target organ for both routes of administration (organ still never described in the literature for the toxic effect of I3C at high doses). In addition, for the first time we used both male and female mice to carry out toxicity studies. This is because, in females, the effect of I3C may be subject to hormonal changes in the metabolism of I and II phases. In fact, this analysis showed a different tolerability dose in males and females to be considered for any future clinical trials in which I3C will be used as an antiviral compound.

Specifically, we employed the mouse in order to evaluate possible toxicity effects of I3C through two different routes of administration: intragastrically (i.g.) and intraperitoneally (i.p.). The estimated LD50 (lethal dose 50%) values in mice were: 1410 and 1759 mg/kg i.g.; while LD50 values for i.p. administration was: 444.5 mg/kg and 375 mg/kg to male and female mice, respectively. We also established that above 1000 mg/kg (i.g.) and 375 mg/kg (i.p.), the toxic effects are characterized by piloerection, dorsal hair dull phenomenon, conjunctival opacification, iritis, and conjunctivitis with reduced spontaneous activity after I3C administration. Moreover, from histopathological examination, we only observed congestion areas in the liver connected to the blood stasis zone, while spleen, kidney, lung and heart did not display gross pathological change after I3C i.g. administration at 2000 mg/kg (i.g.) and 550 mg/kg (i.p.). As reported in [51], the liver represents a fairly large fraction of body weight and appears to be an important reservoir of I3C and related compounds. Below these values, I3C (in particular at 550 mg/kg for i.g. and 250 mg/kg for i.p.) induces neither death nor abnormal toxic symptoms, as well as no histopathological lesions of the tissues, analysed.

In a study conducted by Fletcher et al. [52], it was observed that the athymic mouse model received diets supplemented with 0–100 μmol I3C/g diet for 4 weeks. They found that mice supplemented with I3C were not viable after three days on a 100 μmol I3C/g supplemented diet. On the other hand, mice fed with 10–50 μmol I3C/g supplemented diet survived but showed concentration-dependent adverse effects. Noteworthy, they found intestinal damage occurred in mice that received I3C supplementation as low as 10 μmol/g diet. Therefore, the intestine appeared to be the target of I3C toxicity. Moreover, I3C has been seen to significantly alter the number and width of intestinal villi, which is associated with a dose-dependent reduction in cell proliferation and an increase in apoptosis. Other molecular effects observed for I3C include activation of multiple xenobiotic metabolism pathways. Moreover, this study revealed that the total amount of I3C consumed by an animal per day (5 g at 100 μmol/g) equals to a ~75 mg tablet. One commercially available supplement tablet normally came in the form of 200 mg I3C. This is a concentration comparable to or lower than orally administered doses in humans [53, 54].

In a clinical study, healthy women subjects were given orally up to 1200 mg I3C, and 5 out of 20 subjects reported mild gastrointestinal distress, nausea and vomiting after ingesting a single dose of ≥600 mg I3C [53]. These subjects spontaneously recovered upon discontinuing the supplement with no long-term effects [53]. Few I3C studies reported adverse reactions ranging from skin rash, and a slight increase in gastrointestinal motility, to mild bowel upset [55], indicating the potential safety concern of I3C supplementation.

Similarly, Wong and colleagues [56] enrolled 60 women in a placebo-controlled, double-blind dose-ranging chemoprevention study of indole-3-carbinol (I3C). Each woman took a placebo capsule or an I3C capsule daily for a total of 4 weeks. Participants were given different doses of I3C, between 50 (low-dose) and 400 mg (high-dose). Except for those with a prior history of elevated alanine aminotransferase, none of the participants experienced toxic effects.

In our in vivo experiment, we also observed a different response in male and female mice when treated with different dosages of I3C. These differences can be explained by the fact that I3C is known to exert an anti-estrogenic effect, and female animals may respond to I3C differently due to the interaction between estrogen receptor, and the phase I and phase II metabolism [57–59].

On the basis of the data presented here and what is reported in the literature referring to toxicity studies on I3C and, importantly, keeping into account that a 16.67 μM concentration is equivalent to a 2.5 mg/kg dosing in vivo, we are led to conclude that the concentrations at which the antiviral effects were observed in vitro would be well tolerated in vivo. In conclusion, I3C exhibits a significant antiviral activity, and no toxic effects were recorded for this compound at the indicated doses, characterizing it as a safe and potentially antiviral compound.

Understanding the molecular interactions that modulate the output and egress of viral particles also offers an important opportunity to identify novel host targets for the development of antivirals to prevent and treat infections with coronaviruses and other emerging respiratory viruses [60]. Indeed, host-targeted antiviral therapy may be advantageous to counteract viral resistance and to develop broad-spectrum antivirals. The enzymatic activity of the HECT-E3 ligases has been implicated in the cell egress phase of some RNA viruses possibly hijacking the Endosomal Sorting Complexes Required for Transport (ESCRT) machinery and can therefore constitute a valid target for new classes of antiviral drugs. Interestingly, using a novel bioinformatic approach we have obtained evidence of probable interactions between Nsp15-NendoU endoribonuclease of the SARS-CoV-2 virus with both WWP1 and NEDD4, both physically and functionally (Novelli G et al., unpublished). NendoU activity of Nsp15 is responsible for the protein ability to interfere with interference with the innate immune response [61]. Nsp15 degrades viral RNA to hide it from the host defences [62]. Nsp7b also interacts with NEDD4L with evidence (level = 2) (Novelli G et al., unpublished) [63]. NSP12 is a component of coronaviral replication and transcription machinery, and it appears to be a primary target for the antiviral drug remdesivir [47].

Overall, this study demonstrated for the first time that I3C has an anti-SARS-CoV-2 effect independently of the time of treatment in respect to the time of viral infection. I3C also proves effective against the SARS-CoV-2 Omicron variant. Moreover, we provided evidence of the toxicology effects of this compound in animal models setting and although further studies will be needed to assess the antiviral activity of I3C on in vivo model, it appears to be a promising candidate for its use in human clinical trials. On the whole, I3C is a promising antiviral candidate for the treatment of SARS-CoV-2 infection.

## Materials and methods

### Cells

Vero E6 cells are kidney epithelial cells originally extracted from an African green monkey (*Chlorocebus sp*.; formerly called *Cercopithecus aethiops*). Cells were maintained in Minimum Essential Medium (MEM), supplemented with heat-inactivated 10% foetal bovine serum (FBS), 2 mM L-glutamine and 1% penicillin/streptomycin solution (Sigma-Aldrich, Cat.No. R0883; F7524; G7513; P0781, respectively) and maintained at 5% CO_2_, 37 °C.

### Chemical treatment

Indole-3-carbinol (I3C) was obtained from Sigma-Aldrich (Product Number: 17256, CAS-No.: 700-06-1). For in vitro assays, I3C was dissolved in 100% DMSO and added to the cell culture medium at different concentrations. For in vivo assay, the corresponding dose of I3C suspension is prepared using 10% DMSO, and the solvent was 0.9% sodium chloride solution.

### I3C antiviral test

The antiviral activity of I3C was tested by the SARS-CoV-2-induced cytopathic effect (CPE) inhibition assay using Vero E6 cells infected with the SARS-CoV-2 strain isolated at INMI L. Spallanzani IRCCS (2019-nCoV/Italy-INMI1; GenBank MT06615656) as reported [40]. Briefly, cell monolayers growing in 96-well plates (3 × 10^4^ cells/well) were treated with different doses of either I3C or DMSO according to three different protocols: (i) pre-treated for 1 h before infection (pre-treatment); (ii) at the same time of infection (co-treatment); 1 h after infection (pre-treatment). DMSO was used as control since I3C is solubilized in this compound. Cells were infected at 0.001 multiplicity of infection (MOI; which reflects the ratio of PFU to the number of infected cells), using MEM supplemented with heat-inactivated 2% FBS and 2 mM L-glutamine. In the following 72 h, cells were treated by adding the compound/control to the culture medium every 24 h and maintained at 37 °C with 5% CO_2_. At 72 h post-infection, supernatants were discarded and 100 µL of crystal violet solution (Merck Life Science, Milan, Italy; Cat. No. 9448-2.5L-F) containing 2% formaldehyde (Carlo Erba reagents, Milan, Italy; Cat. No. 415666) were added to each well for 20 min. Subsequently, the fixing solution was removed, plates washed with tap water and then immersed in a bath of 2% formaldehyde solution in PBS for further 20 min. Finally, cell viability was evaluated with a photometer measuring the optical density (OD) at 595 nm and reported as the percentage of surviving cells compared to the uninfected cells. Results are expressed as the mean ± SD. Statistical analysis of data was performed using Wilcoxon test and analysed using Graph Pad (GraphPad Prism 9).

### Pseudotypes SARS-2-S infection and I3C treatment in hLORGs

For treatments with I3C, the hLORGs [42] were incubated for a minimum of 1 h with the I3C at 16.7 µM and then the VSVpp.SARS-2-S virus (D614G) was added to the organoids (previously disrupted into small clumps) and left to act for 4 h at 37 °C. hLORGs are then incorporated in drops of Matrigel GFR at a cell density of ~1200–1600 cells/μL, using CK + DCI media with Y-27832 and left to grow for 72 h and then analysed (as a reported in [42]). In particular, I3C was added at different time points as described in [40] and specifically 1 h before and 4, 24 and 48 h after pseudovirus infection. The infection with the SARS-CoV-2 pseudovirus containing eGFP and gene expression analysis were evaluated at 72 h post-treatment. Briefly, Trizol Reagent (Invitrogen Life Technologies Corporation, Carlsbad, CA, USA) was used to extract total RNA from cells, according to the manufacturer’s instructions. Total RNA samples were treated with DNase I-RNase-free (Ambion, Life Technologies Corporation, Foster City, CA, USA) to remove genomic DNA contamination. One µg of RNA was reverse transcribed and used in RT-qPCR using the Life Technologies Corporation’s High-Capacity cDNA Archive kit (Foster City, CA, USA). SYBR Green was used to assay mRNAs (Life Technologies Corporation, Foster City, CA, USA). As reference genes, 5.8S and GAPDH were employed. Primer sequences will be given upon request. The comparative DDCt methods were used to quantify relative gene expression levels.

The pseudovirus used in this experiment was kindly gifted by Hoffmann lab, German Primate Center–Leibniz Institute for Primate Research, Gottingen, Germany. Briefly, the Vesicular Stomatitis Virus (VSV) pseudovirus system was employed to produce the SARS-CoV-2 pseudovirus, which displays the SARS-CoV-2 spike protein (S) on the VSV particle surface. The replication-deficient VSV vector that lacks the genetic information for VSV-G and instead codes for two reporter proteins, enhanced green florescent protein (GFP) and firefly luciferase (Fluc) (VSV*∆G-Fluc) to generate SARS-CoV-2 S-pseudotyped particles, that accurately mimics key aspects of SARS-CoV-2 entry into cells. The efficiency of virus entry was first observed by evaluating GFP fluorescence (data not shown) and after was specifically quantified by performing luciferase assay. The VSV pseudo virus replicates in 16 h and without its original G is restricted to a single round of replication.

### I3C treatment against Omicron variant

Vero E6 cell monolayers growing in 96-well plates (3 × 10^4^ cells/well) were treated for 1 h with 16.7 µM of I3C before Pseudotype Lentivirus SARS-2-Omicron (ReVacc Scientific) infection. This pseudotype virus uses recombinant lentivirus to carry spike protein of SARS-CoV-2 (GenBank: MN908947) with multiple mutations initially identified in variant of Omicron (B.1.1.529) (BA.1). As pseudo lentivirus infectivity without its original envelope glycoproteins G is restricted to a single round of replication. Cell infection can be monitored by luciferase activity. DMSO was used as uninfected control since I3C is solubilized in this compound. Cells were infected at 250 ffu/well using MEM supplemented with heat inactivated 2% FBS and 1% L-glutamine in the presence of I3C and DMSO treatments. After 1 h of incubation, the viral input was replaced by fresh medium containing either I3C or DMSO. Cells were then treated with either I3C or DMSO every 24 h and incubated at 37 °C with 5% CO_2_ for 60–72 h, when percentage of infected cell was measured by luciferase assay.

### Animal

50 male and 50 female BALB/c mice (4–6 weeks of age; body weight of 18–22 g) were obtained from Beijing Charles River Experimental Animal Center. All mice were kept in SPF-animal facility (Laboratory Animal Usage License Number of Testing Facility: SYXK (SU) 2020-0028). The mice are individually housed in cages elevated off the floor supplied by contract vendor. These cages conform to standards set forth by the US Animal Welfare Act. The cage size complies with the recommendations set by the Guide for the Care and Use of Laboratory Animals. Each mouse, at the commencement of its dosing, was aged between 4 and 6 weeks old and its weight decreased in an interval within ±20% of the mean weight of any previously dosed animals.

### Assessment of acute preclinical toxicity in mice

Based on the practice guide (http://www.fda.gov/cder/guidance/index.htm) for the dose conversion between animals and human and OECD Test Guideline 425 (Acute Oral Toxicity: Up-and-Down Procedure), 50 mg/kg of I3C was considered the optimal starting dosage for acute toxicity test of I3C in mice. The dosing range of I3C was considered from 50 to 2000 mg/kg (The default dose level is 3.2). The test substance was administered in a single dose by gavage using a stomach tube (20 mL/kg). The mice should be fasted prior to dosing (3–4 h). Following the period of fasting, the mice were weighed, and the test substance administered. After the I3C has been administered, food withheld for 1 h in mice. I3C was tested using a stepwise procedure, each step using three mice. The dose (50 mg/kg) of the first mouse is one lever lower than starting dose. If the animal was alive, a higher dose is given to the second mouse, and if the first mouse is dead or dying, then a lower dose is given to the second mouse.

Mice were divided into different groups (*n* = 3 females and male/group). The control group received vehicle alone, and I3C was tested in single doses of 2000, 1750, 1500, 1000 and 550 mg/kg intragastric (i.g.) and 1000, 550, 375 and 250 mg/kg intraperitoneal (i.p.) route. The dose responsible for the death of 50% of the experimental animals (LD50) was estimated. All mice were observed individually for different parameters after I3C i.g. and i.p. dosing at least once during the first 30 min, periodically during the first 24 h, with special attention given during the first 4 h, and daily thereafter, for a total of 14 days.

According to the results of observation 48 h after administration, the time interval between treatment groups was determined by the onset, duration, and severity of toxic signs. Treatment of mice at the next dose was delayed until one is confident of survival of the previously dosed mice. Each living mouse was observed for 14 days, and the later dead mice are recorded as a death in the statistics of the results. If the mouse has no obvious toxicity after administration, the test period was 7 to 10 days. If the mouse has an obvious toxic reaction such as weight loss, it was observed continuously for 14 days after administration, and the test period is about 28 days.

The mice in the treatment group and the control group were evaluated according to the following indicators: toxic dose (LD50, death-dose curve, 95% confidence limit); symptoms; weights and histopathology. The pain score index was evaluated after I3C administration according to scoring standard of pain index for experimental animals as follow: 0: Normal hair and activity of the experimental animals; 1: part of the hair of the experimental animals was erect and temporarily arched back; 2: the fur of the experimental animals was obviously rough and intermittent arched back; 3: the fur was obviously rough, accompanied by other symptoms such as arched back, slow reaction and behaviour, and even death).

### Histopathology

For histological analysis, the liver, heart, spleen, lung and kidney were fixed in 4% formalin followed by dehydration, paraffin embedding, sectioning, and standard Haematoxylin&Eosin (H&E) staining. All organs paraffin sections were viewed by light microscopy and carefully examined the number of congestions containing red blood cells in each section after both I3C i.g. and i.p. by a pathologist and marked directly on the representative H&E-stained section.

### Statistical analysis

The results are expressed as the mean ± SD. The data are sorted out in Excel 2017 and SPSS 16.0. Statistical analysis of data was performed using one-way ANOVA test and Kaplan–Meier test by SPSS 16.0 Software. For in vivo acute toxicity, the toxic dose (LD50, death-dose curve lethal dose and 95% PL confidence interval) values were calculated by AOT425 StatPgm Software.

## Data Availability

The corresponding author will provide the original data used to support the findings of this study upon reasonable request.
